# Developing Digital Facilitation of Assessments in the Absence of an Interpreter: Participatory Design and Feasibility Evaluation With Allied Health Groups

**DOI:** 10.2196/formative.8032

**Published:** 2018-01-09

**Authors:** Jill Freyne, Dana Bradford, Courtney Pocock, David Silvera-Tawil, Karen Harrap, Sally Brinkmann

**Affiliations:** ^1^ Australian eHealth Research Centre Commonwealth Scientific and Industrial Research Organisation Marsfield Australia; ^2^ Australian eHealth Research Centre Commonwealth Scientific and Industrial Research Organisation Pullenvale Australia; ^3^ Western Health Melbourne Australia; ^4^ Australian eHealth Research Centre Commonwealth Scientific and Industrial Research Organisation Herston Australia

**Keywords:** mobile apps, cultural diversity, culturally appropriate technology, cross-cultural care, language barriers, health care delivery, ehealth allied health

## Abstract

**Background:**

To ensure appropriate and timely care, interpreters are often required to aid communication between clinicians and patients from non-English speaking backgrounds. In a hospital environment, where care is delivered 24 hours a day, interpreters are not always available. Subsequently, culturally and linguistically diverse (CALD) patients are sometimes unable to access timely assessment because of clinicians’ inability to communicate directly with them.

**Objective:**

The aim of this study was to design and evaluate CALD Assist, a tablet app to assist communication between patients and allied health clinicians in the absence of an interpreter. CALD Assist uses key phrases translated into common languages and uses pictorial, written, and voice-over prompts to facilitate communication during basic patient assessment.

**Methods:**

CALD Assist’s design, functionality, and content were determined through focus groups with clinicians and informed by interpreting and cultural services. An evaluation was conducted in a live trial phase on eight wards across 2 campuses of a hospital in Victoria, Australia.

**Results:**

A commercial grade CALD Assist mobile app for five disciplines within allied health was developed and evaluated. The app includes a total of 95 phrases in ten different languages to assist clinicians during their initial assessment. Evaluation results show that clinicians’ confidence in their assessment increased with use of the CALD Assist app: clinicians’ reports of “complete confidence” increased from 10% (3/30) to 42% (5/12), and assessment reports of “no confidence” decreased from 57% (17/30) to 17% (2/12). Average time required to complete an assessment with patients from non-English speaking backgrounds reduced from 42.0 to 15.6 min.

**Conclusions:**

Through the use of CALD Assist, clinician confidence in communicating with patients from non-English speaking backgrounds in the absence of an interpreter increased, providing patients from non-English speaking backgrounds with timely initial assessments and subsequent care in line with their English speaking peers. Additionally, the inclusion of images and video demonstrations in CALD Assist increased the ability to communicate with patients and overcome literacy-related barriers. Although a number of hurdles were faced, user uptake and satisfaction were positive, and the app is now available in the Apple App Store.

## Introduction

### Background

Good communication in clinical settings is the key to avoiding medical mishaps [1,2]. Clinicians must obtain and communicate accurate information to patients to complete assessments and provide care. It is critical that information being communicated by a patient to a clinician is accurate and complete. Similarly, it is crucial that clinicians can communicate effectively and accurately with patients to inform them of risks and to provide education.

In acute hospital settings, assessment delays for culturally and linguistically diverse (CALD) patients are common, as clinicians require interpreter services and for a variety of reasons services may not be immediately available [3]. The requirement for accurate interpreting is never more crucial than in the medical domain. Delays in assessments can place patients at risk of dehydration, choking, falls, wound infection, and poor quality of life. For example, dysphagia is common after stroke, and early identification is important because of potential aspiration risk and to determine patients’ suitability for oral feeding [4].

The traditional approach to ensuring patient safety and appropriate communication in clinical settings has been to use professional interpreters. This model has worked with much success, but as human migration increases and health service budgets are placed under increasing pressure, interpreters are not always available to assist in a timely manner. Interpreter demand is unsurprising given that communities served by Australian hospitals vary in cultural diversity, with some hospitals serving areas where more than 150 languages are spoken. Non-English speaking patients are sometimes unable to access timely assessment, causing inequity in service delivery and often frustration and anxiety for patients, their carers, and clinical staff.

### The Use of Mobile Interpreter Technology in the Clinical Setting

Mobile technology has been recognized as a potential solution to interpreter availability, with Web-based tools and apps available for use. A flexible Web-based tool for translation is Google Translate [5]. Google Translate allows clinicians to type in any phrase and receive a text and audio translation in 91 languages. Google Translate has two major drawbacks for use in the medical domain: (1) clinicians are time-poor, and (2) the requirement to type phrase after phrase to receive a translation is impractical. More worryingly, Google Translate has varying levels of accuracy depending on language [6], with low accuracies reported for even simple medical terminology. A recent study in which ten phrases were translated into 26 languages showed that only 57.7% of phrases were accurately translated [7]. Errors included a mistranslation of “your child is fitting” to “your child is dead.” Low translation accuracy in serious health situations will at minimum cause distress and potentially harm.

A number of other health information translation apps, which contain libraries of phrases and translations, are now available. These include MediBabble [8], Canopy Medical Translator [9], and xprompt [10]. MediBabble is currently available in five languages, Canopy Medical Translator is available in 15 languages, and xprompt is available in 22 spoken languages and two sign languages. MediBabble and Canopy Medical Translator contain text and audio translations of extensive lists of questions and phrases covering topics such as medication, allergies, medical history, and current complaints. Xprompt, additionally contains video sequences for the two sign languages. To our knowledge, the only app that has been evaluated in a clinical setting is xprompt, with participants generally supporting the introduction of mobile apps to support communicating with foreign language patients but not very enthusiastic about the app’s practical functionalities [10].

All four cited apps use text and audio to communicate the output of their translations, but evidence from cultural advocacy groups shows that CALD information should be available in a variety of formats including audio-visual and pictorial resources [11]. The requirements for multimodal formats are multifactorial. First, CALD groups have been shown to have low literacy levels, even in their first languages [11]. Second, CALD patients tend to be older and have age-associated vision and hearing decline [12], limiting the effectiveness of small text and low-grade audio.

Additionally, whereas MediBabble, Canopy and xprompt include questions and phrases for clinicians to communicate with patients, there is no functionality for the patient to respond effectively to the questions or instructions if they do not have proficient verbal skills in English. The omission of responses for questions with simple yes or no answers can be overlooked, but when clinicians require responses to open questions such as “What type of walking aid do you use?” or “How long have you been experiencing pain?” existing apps do not assist patients in responding. This scenario is likely to frustrate the patient and the clinician.

### Introducing CALD Assist as a Novel Communication App

This paper reports the design, development, and feasibility evaluation of CALD Assist, a novel user-centric communication app designed to support assessments with CALD patients when an interpreter is not present. To inform the app’s design and content, we conducted a user needs analysis with clinicians from five allied health groups. To assess feasibility of the resulting app under hospital conditions, the app was trialed for 6 months in a controlled introduction at Western Health, a hospital in Victoria, Australia.

Evaluation results demonstrate that a mobile app can effectively be used to assist allied health clinicians during their initial assessments with patients from non-English speaking backgrounds. By using the CALD Assist app, clinician confidence during initial assessments of patients from non-English speaking backgrounds increased, whereas the time required to complete an assessment in the absence of an interpreter reduced.

## Methods

### Design

A user needs analysis was undertaken to inform the content, design, and structure of the CALD Assist app. Two components were undertaken as part of the user needs analysis: (1) a review into the languages required to be part of app and (2) focus groups conducted to elicit information from allied health end users on their current practices and modes of assessment [10].

After the final development of the app, a feasibility evaluation based on a comparative (before and after) study was conducted to quantify the value provided by CALD Assist in assisting allied health clinicians to complete assessments with patients from non-English speaking backgrounds when an interpreter was not available. Specifically, we aimed to determine (1) staff and patient acceptance and satisfaction levels and (2) efficacy of the iPad app. The evaluation was divided in two stages: a baseline data collection stage and a live trial.

All stages of the project included in the user needs analysis and feasibility evaluation were conducted with ethics approval from Western Health Low Risk Human Research Ethics Committee (LNR/14/WH/143).

### Participants

Inclusion criteria for clinician participation for both components of the study required participants to be employees of Western Health, working as allied health clinicians within physiotherapy, occupational therapy (OT), speech pathology, dietetics, or podiatry.

Recruitment of user needs focus group participants was achieved through the managers of allied health discipline. Each identified staff member was invited by email. A total of 19 staff members participated.

The feasibility evaluation included clinician and patient participant groups. All focus group participants were invited and agreed to participate in the baseline data collection stage of the evaluation. Additionally, all allied health staff working on wards where the CALD Assist app was introduced were invited to participate in the live trial. Candidate clinical participants were contacted via an invitation email that included a copy of the information sheet and consent form. Clinician training was conducted by the principal investigator (PI) at designated allied health discipline staff meetings. A total of 58 clinician participants were recruited to the trial, including the 19 participants who also consented for the focus groups and baseline data collection. As opportunity to participate was dependent on a need for the app on trial wards during the trial period; not all clinicians were able to take part.

During the live trial, non-English speaking patients attending a follow-up assessment were invited to provide feedback with an interpreter present. Consent was obtained through the interpreter. One patient participant was recruited.

### Procedure

User needs focus groups lasted up to 90 min, and each group was attended by clinicians from a single allied health discipline. Each focus group was audiorecorded. Aspects under discussion included the type of patient typically assessed, the timing of the assessments in relation to the patient journey (on admission, discharge, etc), the duration, outcomes and implications of the assessments, the phrases or instructions used during an assessment, the assessment setting, and the broad challenges seen to impact on the successful integration of the app into standard care in the inpatient setting.

As a follow-up exercise, participants were asked to identify phrases suitable for their app-enabled assessments. These were provided to the researchers in the week following the focus groups. The phrase lists supplied during and after the focus groups were aggregated, refined, and classified in relevant subgroups by the project team before being circulated to participants and colleagues for discussion and approval.

The feasibility evaluation was based on a comparative (before and after) study conducted on eight wards across 2 campuses of Western Health, in Victoria, Australia. Wards comprised acute aged care, acute orthopedics and neurosurgery, general medicine, oncology and gastroenterology, surgical, respiratory, and neurology. Baseline data were collected by clinicians conducting assessments on CALD patients in the absence of the app over a period of 3 months. These data pertain to information about the patient and the nature and duration of the assessment. Following the baseline data collection, a live trial was conducted over a 23-week period. The trial commenced in February 2015, with the CALD Assist app being introduced on four wards and expanded in June 2015 to include four additional wards.

Software embedded in the CALD Assist app captured interaction logs showing all app usage during the live trial. Additionally, qualitative data were sought from primary and secondary app users, clinicians, and patients, respectively, and was captured through 3 separate questionnaires:

1. *The post assessment questionnaire,* which clinicians had the option to complete after they conducted an assessment using the CALD Assist app. This data describe the nature and duration of the assessment, as well as basic information about the patient to determine whether the app was successful in facilitating assessments and improving communication between clinicians and patients.
2. *The participant feedback questionnaire (staff)*, completed on the Web by clinical staff at the end point of the trial. A link to this questionnaire was emailed to all clinician participants upon completion of the study. The survey captured details of clinician experiences with the app.
3. *The participant feedback questionnaire (patient)*, completed by the PI through an interpreter. The survey captured feedback from patients who experienced an assessment with the app. Responses to the questions were recorded by the PI present.

### Data Analysis

Qualitative and qualitative analysis was used. All audio recordings were transcribed and analyzed by themes using NVivo (QSR International). Data logs were analyzed using Java (Oracle Corporation), and quantitative data resulting from questionnaires were analyzed using descriptive statistics.

## Results

This section presents the results of the user needs analysis­ (language identification, focus groups, and app design) and the feasibility evaluation (app log analysis, baseline data, and live trial).

### Languages Identified for Inclusion

A review of the 2011 Australian Census and Australian Bureau of Statistics data showed that 19% of Australians did not speak English at home. The most common languages spoken at home, excluding English, were Mandarin (1.7%), Italian (1.5%), Arabic (1.4%), Cantonese (1.3%), and Greek (1.3%). The community serviced by Western Health in Victoria is one of the most culturally diverse communities in Australia, with 38% speaking a language other than English at home, totaling over 150 languages and dialects.

In consultation with the Western Health language services manager, the number of occasions of interpreting service at Western Health were analyzed. The ten most common languages serviced by the Western Health language services were Mandarin, Cantonese, Vietnamese, Italian, Greek, Macedonian, Serbian, Croatian, Arabic, and Spanish, largely reflecting the common languages identified in the 2011 Australian Census. These ten languages were identified for inclusion in CALD Assist. Although the most unmet need at Western Health was for specific dialects such as Chin Hakka, the unmet need of this language was outweighed compared with the overall need of the most common languages.

### Focus Groups: Understanding the Assessment Context and Content Requirements

Allied health assessment results inform treatment teams on manual handling, communication, and dietary plans, in addition to medical care plans. Delays in the provision of care and in discharging patients can increase the patient’s length of stay, which inconveniences the patient and increases the cost of care.

Allied health assessments are usually conducted at the patient’s bedside. Clinicians typically sit at the head of the bed and suggest that an iPad or similar device could be placed on a patient’s table. Podiatrists differ from other allied health disciplines in that their assessment is conducted from the bottom of the bed as they examine the patient’s feet. Podiatrists wear surgical gloves while conducting assessments and hold equipment such as scalpels. Thus, podiatrists identified the need for infection control procedures for use of the iPad and protocols to be introduced to ensure patient and clinician safety if both handling the device and their equipment. When questioned about suitable access to the intended CALD Assist app, all clinicians indicated a preference for the app to be available on each ward. A suggestion was made by a podiatrist that the podiatry app could be located with the equipment that they carry to the patient’s bedside.

Participants noted that in situations where an assessment of a CALD patient is required, but when an interpreter is unavailable, clinicians are resourceful and often attempt to gain some information from patients through the use of gesture and demonstration.

Physiotherapy, OT, and dietetic assessments require the patient to answer closed questions about their current, and often past, health status. Physiotherapy and speech pathology clinicians also observe patients doing actions such as walking, getting out of bed, climbing stairs, coughing, or swallowing as part of an assessment. The nature of the closed questions and instructions for physical assessments are well suited to a two-way communication app.

OT assessments are highly structured and gather detailed information on activities of daily living and the set-up of patient homes. Dieticians often complete a nutrition assessment that requires responses to open-ended questions. The detailed nature of both of these assessments limits the applicability of a full OT or dietetics assessment for this app, but participants agreed that an app could ascertain some useful basic information while waiting for a full assessment, or in determining if a full assessment is required.

Podiatrists assess patient’s feet, and podiatry assessments typically include some treatment (eg, lancing a wound or debriding) that requires use of instruments such as scalpels and as such is more invasive than other discipline assessments. Thus, including the ability to gain consent from patients for podiatry input and being able to explain each stage of the podiatry assessment and intervention process to the patient were important factors.

All clinicians suggested that the app would facilitate broader communication than simply the assessment content. Participants noted the need for clinicians to be able to introduce themselves and to explain a little about the assessment and its purpose. Similarly, being able to exit an assessment, explain next steps, or inform the patient that they will return were considered important in increasing patient comfort and experience.

Participants also noted the value of being able to provide education or strategies to patients around precautions that they should be taking, given their conditions. These included safety precautions for a patients’ time in hospital, such as not walking to the bathroom unaccompanied, eating slowly, and keeping wounds dry, and recommendations for use of equipment at home once discharged. Dieticians, speech pathologists, and OTs noted a desire to be able to show patients images to ascertain knowledge about preferences for food and drink, to communicate instructions, and equipment used at home. Speech pathologists and OTs suggested video content to demonstrate appropriate swallowing and movements. Further information on the focus groups is provided in the study by Albrecht et al. [10].

### App Design Overview

**Figure 1:**
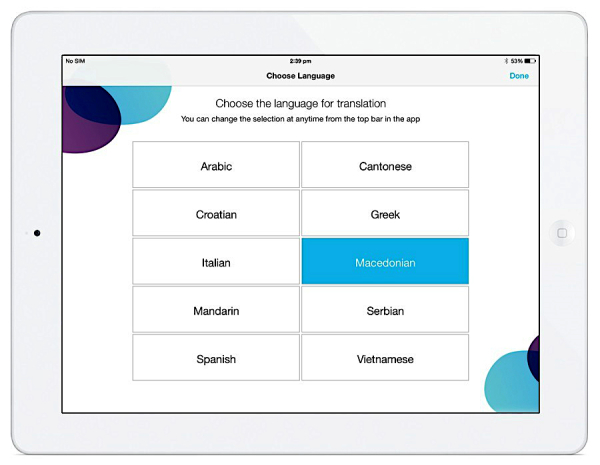
CALD Assist language selection.

**Figure 2:**
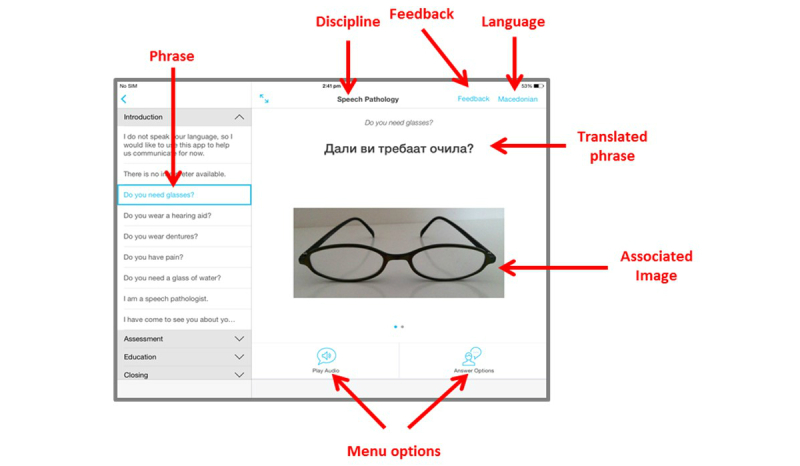
CALD Assist app overview.

On the basis of the outcomes of the user needs focus groups, CALD Assist was developed to be simple in design and function. To use the app in an assessment, the allied health clinician selects a language for use from the ten languages listed (Figure 1). The clinician then selects the group of phrases that they wish to access, grouped by discipline, and the type of phrase that they wish to use, grouped by section (Figure 2). Users can also search for a phrase using keywords. All phrases were translated using a professional translation service and reviewed by professional interpreters employed at Western Health. Audio for each phrase was recorded by the Western Health interpreters.

A total of four sections containing different types of phrases were identified for each discipline: introduction, assessment, education, and closing. A *general phrases* subsection was added to provide simple access to generic phrases for all disciplines, such as “Do you need glasses?” or “Do you have pain?” A sample of the phrases requested by the speech pathology participants is shown in Table 1.

Upon selection of an individual phrase, the content (translated text and appropriate images or video) are displayed on the screen and can be shown to a patient (Figure 2). The menu options allow the clinician to play the audio for the phrase through the built-in iPad speaker. For many questions, gesture-based responses are expected from patients. Where the question has specific answers that cannot be conveyed through gesture, the clinician can display some “answer options” that include text and images. Where a phrase has follow up questions, these can also be accessed through the on-screen menu. All images can be enlarged, and users can swipe between images when more than one image or video is associated with a phrase.

For evaluation purposes, when a clinician completes an assessment, they can select the feedback option on screen. This brings them to the post assessment questionnaire where they can record their experiences using the app. CALD Assist was built as an iPhone operating system (iOS, Apple Inc) 8 compatible iPad app.

**Table 1:** Sample phrases from speech pathology participants.

Section	Phrase
Introduction	I am a speech pathologist.
	I don’t speak your language, so I’d like to use this app to help us communicate for now.
Assessment	Do you cough when you eat or drink?
	Point to where the food is getting stuck.
Education	Sit up when you eat and drink.
	Eat and drink slowly.
Closing	I will return with an interpreter.

### Baseline Data

A total of 45 assessment records of CALD patients were obtained by clinicians, with a balance of male (n=25) and female (n=20) patients, although not all clinicians answered all questions. The majority of patients (98%, 44/45) required an interpreter. The average age of patients requiring interpreters was 75.6 (standard deviation [SD] 15.5), with the youngest patient being 27 years and the oldest being 95 years. The reported time to complete assessments was 41.9 min (SD 16.39) (84% [38/45] of clinicians responded). In line with published data [11,12], participants noted that older CALD patients tended to have age-related sight or hearing impairments that needed consideration, and they may not be literate in their native language.

When clinicians were asked about their confidence that patients’ understood their questions or instructions, 30 of 45 responded. Of these 30, 57% (17/30) responded that they had no confidence, 33% (10/30) responded that they had moderate confidence, and 10% (3/30) that they had complete confidence. It is noted that those reporting complete confidence had the patients’ family member in the room assisting with communication. Clinicians reported that only 50% (20/40 responses) of assessments were completed, with 24 clinicians giving reasons for non-completion including language barriers as the main barrier to completion (67%, 16/24), followed by health of the patient (13%, 3/24), and other (21%, 5/24).

CALD Assist’s inbuilt logging captured all usage data during the live trial. A single session was defined as an episode of use, where at least one phrase is selected. Given the unsupervised nature of app usage, it is not possible to distinguish between app familiarity sessions and usage in real assessment sessions. Considering that as all participants were provided with training where they had access to the app before the start of the trial, we propose that the activity captured during the trial phase corresponds to usage in assessment contexts. A total of 32 sessions were captured in 23 weeks, indicating that CALD Assist was used to perform an assessment in the absence of an interpreter on average once a week.

Table 2 shows the uptake of CALD Assist on each of the eight wards on which it was deployed. The first four wards had iPads with CALD Assist supplied for 23 weeks, the remaining for 6 weeks. Equivalent usage levels were recorded in the wards included in the initial rollout, with each ward conducting between six and nine assessments in total, or between 0.26 and 0.36 uses per week. The length of session use varies between wards but SD is high, indicating a range of assessment times across each ward.

**Figure 3:**
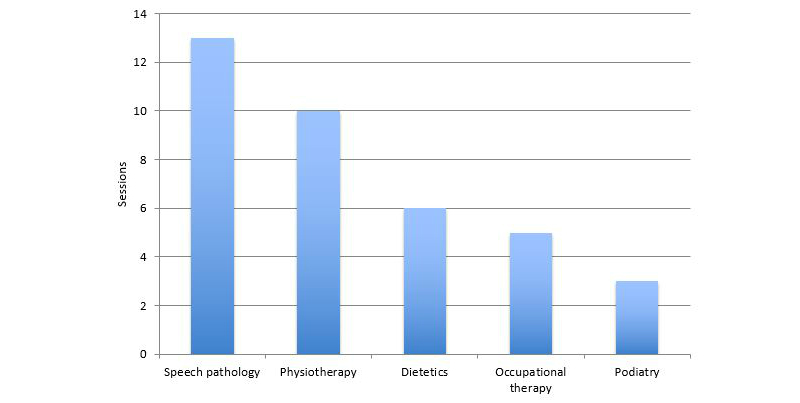
Sessions per discipline. Note that more than one discipline could have been accessed in a single session, according to the clinician needs. As a result, this figure represents more than the 32 total sessions.

The app was used to provide support in a range of languages, with the most frequently selected being Greek, used in 27% (7/26) of the sessions. Vietnamese (19%, 5/26) and Cantonese (15%, 4/26) were also popular, followed by Italian (12%, 3/26), Mandarin (8%, 2/26), and Croatian (8%, 2/26). Spanish, Serbian, and Arabic were each used one single time, whereas Macedonian was not used at all. Language data for the remaining 6 sessions is unavailable. Uptake of CALD Assist varied by clinician type. Speech pathologists used the app more frequently than other disciplines, with 13 sessions recorded (Figure 3). High uptake was also recorded by physiotherapists (ten sessions). Usage by dietetics, OT, and podiatry was low with less than six sessions recorded each.

Of the 95 phrases included in the app, 54 were used during the trial. The most frequently used phrase in the library was a phrase used to introduce the app to patients: “ *I don’t speak your language, so I’d like to use this app to help us communicate for now.”* This phrase was used twice as often as the next most popular phrase: “ *Do you have pain?”* High usage of phrases used to explain the absence of the interpreter, “ *There is no interpreter available,”* and to introduce clinician groups, “ *I am a speech pathologist”* and “ *I am an occupational therapist,”* are noted.

**Table 2:** CALD Assist usage during the trial period.

Trial period and ward		Sessions	Clicks	Duration (min) Mean (standard deviation)
**Usage: 23 weeks**				
	Acute aged care	8	97	12.12 (16.99)
	General medicine	6	36	6.00 (7.92)
	Acute orthopedics and neurosurgery	6	83	13.83 (14.67)
	Oncology and gastroenterology	9	67	7.44 (7.72)
**Usage: 6** **weeks**				
	Surgical	1	1	1 (0)
	Neurology	0	0	0
	General medicine	1	0	0
	Respiratory	1	5	5.00 (0)

**Table 3:** CALD Assist top phrase usage. This list includes only the phrases that were used in at least four sessions.

Phrase	Discipline	Phrase type	Sessions
I don’t speak your language so I’d like to use this app to help us communicate for now.	General	Introduction	12
Do you have pain?	General	Assessment	6
There is no interpreter available.	General	Introduction	6
I am a speech pathologist.	Speech pathology	Introduction	6
I have come to see you about your swallowing.	Speech pathology	Introduction	5
Copy me.	Speech pathology	Assessment	5
Do you use a frame or stick?	Physiotherapy	Assessment	5
Do you need help with bathing?	Occupational therapy	Assessment	4
Please swallow.	Speech pathology	Assessment	4
Do you wear dentures?	General	Assessment	4

In total, the 54 phrases were used 154 times. Of the phrases used, 61.6% (95/154) were assessment phrases, 29.2% (45/154) were introduction phrases, 7.1% (11/154) were education phrases, and 1.9% (3/154) were closing phrases. Grouping phrases by discipline shows high use of the speech pathology and OT phrases. General phrases, available in disciplines, were also common (Table 3).

The average number of phrases used in assessments varied with each discipline. A higher number of phrases (6.2 per session) were used in OT sessions, confirming the findings that OT assessments are detailed and require more questions. Speech pathology and physiotherapy used an average of 3.2 and 2.2 phrases per session, respectively.

Examination of the usage logs shows the selection of a phrase is the most popular task completed, followed closely by playing the audio accompanying the phrase (Table 4). We see reasonable use of the swipe between image function, which is used to browse between multiple images relevant to a single phrase. The capability to show follow up phrases and answer options was rarely used. Low use of the search feature and phrase library list was noted.

### Post Assessment Questionnaire

Clinician’s had the opportunity to provide feedback using the feedback option on the screen (Figure 2). A total of 12 feedback questionnaires were provided by staff participants for assessments conducted with 5 male and 7 female patients in the age range of 21 to 94 years (average age=74 years). To identify the potential impact because of the introduction of the CALD Assist app, data from these questionnaires were compared with the data collected during baseline (Table 5).

**Table 4:** Functionality uptake.

Function	Uses	Sessions
Choose phrase	189	21
Play audio	125	17
Swipe image	86	10
Show feedback form	16	14
Phrase image tapped	12	6
Select feedback incomplete option	12	7
Show answer options	12	7
Choose phrase library phrase	7	4
Search	6	3
Do patient survey	4	2
Show follow-up questions	3	2
Choose full screen mode	2	1
Phrase movie tapped	2	2

**Table 5:** Improvement in efficacy in app-enabled assessments compared with standard assessment. Percentages are rounded to the nearest decimal and therefore may not add to 100%. Baseline data were completed by 45 clinicians, not all of whom answered every question. Post-trial data were completed in full by 12 clinicians.

Measures		Baseline	CALD Assist
**Time to complete assessment (mins)**			
	Max	80	30
	Min	10	2
	Mean (standard deviation)	42.97 (16.34)	15.58 (8.61)
**Confidence in assessment, n (%)**			
	No confidence	17 (57)	2 (17)
	Moderate confidence	10 (33)	5 (42)
	Complete confidence	3 (10)	5 (42)
**Completed assessments, n (%)**			
	Yes	20 (50)	6 (50)
	No	20 (50)	6 (50)
**Reason assessment not** **completed, n (%)**			
	Language	16 (67)	0 (0)
	Health of patient	3 (13)	2 (33)
	Participation of patient	0 (0)	4 (66)
	Other	5 (21)	0 (0)
	Interruption	0 (0)	0 (0)
	Phrase not available	0 (0)	0 (0)

Following implementation, improvements were seen in the length of time required to complete assessments, with the average time required falling from 42 min to 15.6 min. The number of noncompleted assessments was consistent before and following app implementation; however, the reason for noncompletion differed markedly. Before app implementation, 16 unfinished assessments (of the 24 for which reasons were given, 67%) could not be completed for reasons related to language, whereas after implementation, no clinicians cited language as a causative factor. Health and participation of patients were issues that were beyond the scope of the app to address.

Importantly, we see that clinician confidence in their assessment increased with use of CALD Assist. Assessments where no confidence was reported decreased from 57% (17/30) to 17% (2/12). Assessments where complete confidence was reported increased almost fourfold from 10% (3/30) to 42% (5/12). This demonstrates that clinicians believe that they are communicating more effectively with patients when using CALD Assist.

### Staff Participant Feedback

Six responses were received from the Web-based *participant feedback questionnaire (staff)* at the end of the trial. Feedback was received from 3 speech pathologists, 2 dieticians, and 1 occupational therapist. Measures included choosing between a pair of descriptors and usability questions informed by discussions with clinicians. All respondents were female, with an average of 7.5 years of clinical experience. The majority of participants used only positive terms to describe the app (Table 6), with only 1 participant using three negative terms: annoying, not enjoyable, and not effective.

There was greater variation in the self-report section of the *participant feedback questionnaire (staff)* (Table 7). In general, most participants agreed or were neutral to phrases about the app’s ease of use, operation, and clarity. One participant disagreed with the statement that it was easy to get the app to do what they wanted. One participant suggested patients appeared to have problems when communicating with the app, 3 people were neutral, and 2 proposed that patients did not have problems when using the app.

The participant questionnaires also included a section for free text feedback; comments include the following:

- Expansion to include further phrases would be ideal.
- Expansion to include further languages would be ideal.
- It would be great to be able to play audio for instructions or questions that have a second lot of options
- Fantastic app and definitely helpful if no interpreter available. At times, difficult to use if patient is significantly cognitively impaired.
- Often would have loved to use it but required language not available on app.
- Access is a big barrier, it would be used more if the app was located more centrally.
- Excellent tool!

**Table 6:** Term options given to participants to describe the app during feedback (n=6).

Term pairs (negative — positive)	Negative respondents	Positive respondents
Unpleasant — pleasant^a^	-	6
Bad — good	-	6
Annoying — supportive^a^	1	5
Not enjoyable — enjoyable	1	5
Not effective — effective^a^	1	5
Useless — useful	-	6
Irritating — likable^a^	-	6
Worthless — valuable	-	6
Boring — exciting^a^	-	6
Ugly — attractive	-	6
Harmful — beneficial	-	6

^a^This subset of pairs of descriptors was used for patient feedback.

**Table 7:** Self-reported function and usability of the app (n=6).

Question	Strongly disagree	Somewhat disagree	Neutral	Somewhat agree	Strongly agree
I found the app easy to use	-	-	1	2	3
I enjoyed using the app	-	-	2	1	3
I found it easy to get the app to do what I wanted	-	1	1	2	2
Learning to operate the app was easy for me	-	-	-	2	4
I found it easy to become skillful at using the app	-	-	2	2	2
My interaction with the app was clear and understandable	-	-	2	3	1
Patients did not appear to have problems when communicating via the app	-	1	3	1	1
The app was useful as a communication tool when interpreters were not present	-	1	2	-	3
All of the phrases that I needed were available in the app^a^	1	1	-	2	1
All of the images or videos that I needed were available in the app^a^	1	1	-	2	1
All of the languages that I needed were available in the app^a^	-	2	-	-	3
The app contain phrases that are appropriate for me to carry out initial assessments^a^	-	1	-	-	4

^a^One participant did not answer this question.

### Patient Participant Feedback

A 74-year-old male who spoke Croatian consented to participate by completing the *participant feedback questionnaire (patient)*. He strongly agreed with all questions assessing effectiveness of the app (I was comfortable with the app being used for my assessment, the iPad app helped me communicate with my therapist and was useful as a communication tool, I understood what the therapist was trying to say, because they used the iPad app, I could clearly hear the audio that the iPad app played, and I could clearly see the screen of the iPad app). When asked to choose descriptive terms for the app (see descriptor pairs in Table 6), he choose four positive terms (pleasant, supportive, effective, and likeable), but when presented with a choice between boring and exciting, he chose boring.

## Discussion

### Principal Findings

Our key findings show that CALD Assist was used, on average, once a week to complete an initial assessment. Through its use, a number of patients from non-English speaking backgrounds received timely initial assessments and subsequent care, in line with their English speaking peers. Thus, CALD Assist can be seen to have contributed to the goal of the delivery of equitable health care. Additionally, CALD Assist was positively accepted by clinicians who reported increased levels of confidence when communicating with non-English speaking patients when they had access to the app.

We note the high utilization of introductory phrases that allow clinicians to introduce themselves, explain their purpose, and that interpreters are unavailable, which was not possible to achieve before CALD Assist. We also note high usage of a variety of assessment phrases used and high usage of the audio and image cues. This domination of assessment phrases is expected, as the main aim of the app is to facilitate assessments. In most sessions, a number of phrases are used in succession rather than a single phrase used in an ad hoc manner. Finally, the app was used more by the speech pathology clinicians and physiotherapists than the other disciplines. This is not surprising as our needs analysis uncovered the detailed nature of assessments in OT and dietetics domains, the complicating factors of a podiatry assessment requiring the wearing of surgical gloves, and the clinician being positioned at the patient’s feet. We note that although podiatry and dietetics did use the app, their assessments typically only included a single phrase. It is possible that these clinicians initiated assessments but were unable to complete them using the app. We expect that through increased familiarity and promotion campaigns, we will see increased usage of CALD Assist in those disciplines.

CALD Assist does not aim to replace interpreters but to assist in communication when interpreters are unavailable. Thus, we note the rise in clinician confidence in communicating with patients in the absence of an interpreter following the introduction of CALD Assist. We have shown that clinicians are more confident that patients have understood their requests and that language is no longer the main barrier to the completion of assessments. Outside of our formal data gathering, clinicians reported several success stories for CALD Assist not captured through our formal data collection procedure.

Through use of CALD Assist, a speech pathologist determined that ear, nose, and throat (ENT) specialist input was required for a patient who was receiving a swallow assessment, and a referral to the ENT team was made immediately. With use of CALD Assist, it was also determined that a follow-up review was not required. The speech pathologist commented that without CALD Assist they would not have been able to determine the need for ENT and would have needed to return to complete a swallow review the following week when an interpreter was available. In this case, appropriate care was determined through use of CALD Assist when a significant wait time for an interpreter was estimated.

On a separate occasion, a speech pathologist reported advantages of using CALD Assist with patients who are cognitively impaired, a situation where we’d expected the app not to be used. The clinician reported that without CALD Assist the patient was easily distracted; however with CALD Assist, the patient was able to easily attend to the instructions and information provided by the speech pathologist. Although cognitive impairment may impact communication in general, but specifically communication with the app, this example shows that not all cognitive impairments are barriers for CALD Assist use and that benefits of focused delivery through an app exist in health care situations. There were also cases where sufficient information was gathered from a patient with CALD Assist to no longer warrant a full assessment with an interpreter, and the research team had repeated requests from clinicians on nontrial wards to gain access to CALD Assist for their patients.

Negative responses to content feedback (Table 6) suggest that now that the app has been used, refinement of the phrases, images, and languages is recommended. Five participants agreed that the app had appropriate content for their assessments, but negative feedback pertaining to the app having all the phrases and languages needed suggest that additional phrases and languages are desirable, and additional images and videos for existing phrases should be considered. This is supported by the comments, several of which relate to potential refinements. Despite the research protocol specifying that an iPad be located on each ward for clinicians to use, one comment suggests that the iPads were not centrally located. This could explain low usage on some wards and would need to be rectified before commercialization. Additionally, 1 participant disagreed with the ease of use of the app, suggesting that a need for additional training or a revision of design may be needed.

It was identified that other languages may have more prominence in other metropolitan catchments of Australia. For example, there is a greater need for Hindi interpreters in southeast Melbourne and a greater need for Aboriginal dialects in Queensland and the Northern Territory. However, there may be reduced generalization when comparing with Australia-wide trends and needs.

Although it is clear that the app improved the delivery of equitable health care for patients through the reduction in consultation times and provided additional benefits from the perspective of the clinicians, only one feedback questionnaire was obtained from patient participants. Although the responses to this questionnaire were generally positive, alternative methods to obtain additional patient feedback would be required in future research to gather a deeper understanding of the patients’ perception of the app that could inform future developmental stages.

### Challenges

Despite receiving mostly positive feedback, the introduction of CALD Assist was not without its challenges. Throughout the project, a number of hurdles were faced that impacted on uptake of the app and the level of feedback received during the trial.

First, the hospital performing the trial underwent significant organizational change immediately before the trial, which included the opening of a new intensive care unit and cardiac care unit and a reorganization of wards or units. This included staff in the 2 campuses included in the trial. It is hypothesized that this significant change in ward, service, and staff location impacted on the use of CALD Assist. Many staff participants were required to relocate to new workplaces or clinical areas, and priority was duly given to meeting organizational strategic priorities rather than implementing a new technology. We saw great levels of enthusiasm in the user needs analysis of the trial, and details on 45 assessments were gathered in the 3-month baseline data gathering phase, illustrating that clinical staff were keen to inform the development of the app and participate in this research before organizational change. Despite a 23-week trial period, only 12 assessment details were recorded, and low responses to feedback questionnaires were received. Second, clinicians are busy people with many patients under their care, and because of ethics constraints, we were unable to directly follow up with participants other than to send a reminder email encouraging feedback.

Third, the introduction of new clinician-focused technology in a hospital environment was also a challenge faced by the project team. Many experienced clinicians reported that they found it challenging to change established behaviors or practices. As a result, they may enter an assessment session without considering CALD Assist as a tool to facilitate assessment. Further promotion of the CALD Assist app will continue during the next 12 months to further embed its use in current practice.

Finally, CALD Assist was used successfully to conduct assessment with many patients. However, it was challenging to collect meaningful feedback about the app directly from patients. It is hypothesized that this was largely because of patients being acutely unwell in an unfamiliar and often overwhelming hospital environment. Obtaining patient feedback proved to be more difficult than expected. Many of the patients who had assessments conducted with the assistance of the CALD Assist were suitably aware for the app to be used effectively for the assessment; however, they were unable to provide feedback during clinical review because of cognitive impairment and an inability to recall the use of the app. The delay between use of the CALD Assist and collection of feedback may have contributed to patient’s recall of the app; however, this delay was largely unavoidable because of the need for an interpreter to assist with the collection of feedback.

### Comparison With Prior Work

CALD Assist is unique in facilitating two-way communication between patients and clinicians. Although MediBabble, Canopy, and xprompt include questions and phrases for clinicians to communicate with patients, there is no functionality for the patient to respond effectively to the questions or instructions if they do not have proficient verbal skills in English. The omission of responses for questions with simple yes or no answers can be overlooked, but when clinicians require responses to open questions, existing apps do not assist patients in responding. Patients’ only response option is to answer in their native language, which the clinician is unlikely to comprehend. This scenario is likely to frustrate the patient and the clinician. CALD Assist provides translations of response options through both images and text. When asked what type of walking aid they have in their home, patients can browse a set of images to identify an aid similar to the one which they own. When asked how long they have been experiencing pain, they can indicate on a timeline of days, months, or weeks annotated in their own language. The ability to seek detailed information from patients through two-way communication is a key advantage in an environment where accuracy is relied upon. We believe that the inclusion of images and video demonstrations in CALD Assist increased the ability to communicate with patients and overcome age- and literacy-related barriers.

As is the case with many mobile apps, neither MediBabble and Canopy nor xprompt provide evidence on their efficacy. Little information is available on the efficacy of mobile technology in the health domain [13-17], which causes reluctance by policy makers and clinicians to include the mobile apps in standard practice. Although not yet clinically evaluated, available technologies suggest that a mobile app may be used to assist patient-clinician communication when interpreters are unavailable; reducing inequity in service delivery, improving staff confidence, and patient care. Nonetheless, a number of gaps need to be addressed before this technology can be effectively used in a clinical setting, including the provision of high grade audio-visual and pictorial resources, a design that is practical and easy to use by clinicians, and allowance for patient responses.

We hope that by providing evidence in the design and evaluation of CALD Assist that we have addressed these gaps and will instill confidence in allied health clinicians in the use of the app as part of their care delivery. We look forward to comparing the patient experience with CALD Assist to other apps in the market in future studies. CALD Assist is available for download to iPads via the Apple App Store.

## Abbreviations

CALD: culturally and linguistically diverse
ENT: ear, nose, and throat
OT: occupational therapy
PI: principal investigator
SD: standard deviation

## References

[1] Sutcliffe KM, Lewton E, Rosenthal MM (2004). Communication failures: an insidious contributor to medical mishaps. Acad Med.

[2] Manojlovich M, Adler-Milstein J, Harrod M, Sales A, Hofer TP, Saint S, Krein SL (2015). The effect of health information technology on health care provider communication: a mixed-method protocol. JMIR Res Protoc.

[3] Smith Ke, Cardinal P, LeBlanc F, Brindley (2014). Improving medical communication: skills for a complex (and multilingual) clinical world. Can Respir J.

[4] Ramsey DJ, Smithard DG, Kalra L (2003). Early assessments of dysphagia and aspiration risk in acute stroke patients. Stroke.

[5] Google Translate.

[6] Chen X, Acosta S, Barry AE (2016). Evaluating the accuracy of Google translate for diabetes education material. JMIR Diabetes.

[7] Patil S, Davies P (2014). Use of Google Translate in medical communication: evaluation of accuracy. Br Med J.

[8] Medibabble.

[9] Canopy.

[10] Albrecht UV, Behrends M, Schmeer R, Matthies HK, von Jan U (2013). Usage of multilingual mobile translation applications in clinical settings. JMIR Mhealth Uhealth.

[11] The Ethnic Communities Council of Victoria (2012). An Investment Not an Expense: Enhancing Health Literacy in Culturally and Linguistically Diverse Communities.

[12] Fozard JS, Gordon-Salant S, Birren JE, Schaie KW (2001). Changes in Vision and Hearing with Aging. Handbook of the Psychology of Aging, 5th edition.

[13] Freyne J, Pocock C, Bradford D, Harrap K, Brinkman S (2015). Designing technology for assessments of CALD patients. Stud Health Technol Inform.

[14] Erickson K (2015). Evidence considerations for mobile devices in the occupational therapy process. OJOT.

[15] Pagoto S, Schneider K, Jojic M, DeBiasse M, Mann D (2013). Evidence-based strategies in weight-loss mobile apps. Am J Prev Med.

[16] Luxton DD, McCann RA, Bush NE, Mishkind MC, Reger GM (2011). mHealth for mental health: integrating smartphone technology in behavioral healthcare. Prof Psychol Res Pr.

[17] Abroms LC, Padmanabhan N, Thaweethai L, Phillips T (2011). iPhone apps for smoking cessation: a content analysis. Am J Prev Med.

